# Dysexecutive difficulty and subtle everyday functional disabilities: the digital Trail Making Test

**DOI:** 10.3389/fneur.2024.1354647

**Published:** 2024-04-03

**Authors:** David J. Libon, Rod Swenson, Sean Tobyne, Ali Jannati, Daniel Schulman, Catherine C. Price, Melissa Lamar, Alvaro Pascual-Leone

**Affiliations:** ^1^Department of Geriatrics and Gerontology, New Institute for Successful Aging, Rowan University-School of Osteopathic Medicine, Stratford, NJ, United States; ^2^Department of Psychology, Rowan University, Glassboro, NJ, United States; ^3^University of North Dakota School of Medicine and Health Sciences, Grand Forks, ND, United States; ^4^Linus Health, Boston, MA, United States; ^5^Department of Neurology, Harvard Medical School, Boston, MA, United States; ^6^Department of Clinical and Health Psychology, University of Florida, Gainesville, FL, United States; ^7^Rush Alzheimer’s Disease Center and the Department of Psychiatry and Behavioral Sciences, Rush University Medical Center, Chicago, IL, United States; ^8^Hinda and Arthur Marcus Institute for Aging Research and Deanna Sidney Wolk Center for Memory Health, and Eleanor and Herbert Bearak Memory Wellness for Life Program, Hebrew Senior Life, Boston, MA, United States

**Keywords:** IADL abilities, mild cognitive impairment, subtle cognitive impairment, executive control, Trail Making Test-Part B, Boston process approach

## Abstract

**Background:**

Digital neuropsychological tests reliably capture real-time, process-based behavior that traditional paper/pencil tests cannot detect, enabling earlier detection of neurodegenerative illness. We assessed relations between informant-based subtle and mild functional decline and process-based features extracted from the digital Trail Making Test-Part B (dTMT-B).

**Methods:**

A total of 321 community-dwelling participants (56.0% female) were assessed with the Functional Activities Questionnaire (FAQ) and the dTMT-B. Three FAQ groups were constructed: FAQ = 0 (unimpaired); FAQ = 1–4 (subtle impairment); FAQ = 5–8 (mild impairment).

**Results:**

Compared to the FAQ-unimpaired group, other groups required longer pauses inside target circles (*p* < 0.050) and produced more total pen strokes to complete the test (*p* < 0.016). FAQ-subtle participants required more time to complete the entire test (*p* < 0.002) and drew individual lines connecting successive target circles slower (*p* < 0.001) than FAQ-unimpaired participants. Lines connecting successive circle targets were less straight among FAQ-mild, compared to FAQ-unimpaired participants (*p* < 0.044). Using stepwise nominal regression (reference group = FAQ-unimpaired), pauses inside target circles classified other participants into their respective groups (*p* < 0.015, respectively). Factor analysis using six dTMT-B variables (oblique rotation) yielded a two-factor solution related to impaired motor/cognitive operations (48.96% variance explained) and faster more efficient motor/cognitive operations (28.88% variance explained).

**Conclusion:**

Digital assessment technology elegantly quantifies occult, nuanced behavior not previously appreciated, operationally defines critical underlying neurocognitive constructs related to functional abilities, and yields selected process-based scores that outperform traditional paper/pencil test scores for participant classification. When brought to scale, the dTMT-B test could be a sensitive tool to detect subtle-to-mild functional deficits in emergent neurodegenerative illnesses.

## Introduction

The currently accepted medical practice to diagnose suspected mild cognitive impairment (MCI) and dementia requires a thorough medical examination and lab tests to rule out unobserved medical problem(s), a brain imaging study, and a comprehensive neuropsychological assessment. Additional information needed in order to characterize MCI and dementia is a care-giver assessment of everyday functional or instrumental activities of daily living (IADL) (1), querying how well patients can manage important everyday activities such as taking medication properly, understanding financial matters, shopping independently, and driving an automobile (2). Among patients with suspected MCI and dementia, problems revolving around episodic memory have traditionally been viewed as an early, if not the first, neurocognitive ability to decline. Nonetheless, recent research underscores an intimate relationship between the presence of dysexecutive behavior, in addition to declining memory abilities, and IADL compromise (3–6). For example, in recent research, Libon et al. (7) administered the Instrumental Activities of Daily Living – Compensation Scale (IADL-C) (8) to memory clinic patients where Jak/Bondi criteria (9, 10) were used to classify participants into MCI subtypes. These researchers reported that, under certain circumstances, declining IADL abilities were, as expected, linked with lower performance on an aggregate, verbal episodic memory index. However, more robust relationships were found linking declining IADL abilities and impaired performance on an executive function index score.

Among the executive tests used by Libon et al. (7) was the Trail Making Test-Part B (TMT-B; Army Individual Test) (11–13) where time to completion was the outcome measure. As is well-known, the Trail Making Test-Part A (TMT-A) asks participants to draw a line connecting numbers inside circles from 1 to 26. The TMT-B requires participants to draw a line starting with the number 1, then the letter A, alternating between numbers and letters until reaching the number 13. Past research suggests time to completion on both Trail Making Tests assess overlapping but different underlying neurocognitive constructs (12). For example, previous reports tend to link visual search and graphomotor information processing speed with Trails A total time to completion (14–16). Past research also links TMT-B time to completion and performance on tests that assess information processing speed (17); however, additional cognitive functions also contribute to successful performance.

TMT-B time to completion has been clearly linked to a wide variety of executive impairments (18–20), including perseverative errors on the Wisconsin Card Sort Test (21), WAIS-III Digit Span Backward test performance (16), and errors made on the clock drawing test (22). Moreover, successful performance on the TMT-B has consistently been shown to be related to relatively intact IADL, functional abilities (23–25). Indeed, the wide number of neurocognitive abilities underlying TMT-B time to completion (14) is a major reason why this test is so sensitive to brain illness.

There is now tremendous interest in the development and deployment of digital cognitive assessments (DCAs) that can be administered with commercial-off-the-shelf mobile devices and automatically scored. As pointed out by Libon et al. (26), advantages of deploying DCAs include standardized test administration and scoring. This mitigates the subjectivity associated with certain test items from traditional cognitive assessments such as the Mini-Mental State Examination (MMSE) and the Montreal Cognitive Assessment sentence production and figure copy test items, respectively (MoCA) (27, 28). Equally important, Libon et al. (26, 29) have demonstrated how DCAs can uncover, measure, and operationally define process-based behavior and neurocognitive constructs previously unobtainable using traditional pencil and paper tests that rely primarily on a single, final score. Indeed, DCA advances include moment-to-moment measures of motor, cognitive, and time-based performance (30–32). Unlike many traditional pen-and-paper or simpler DCAs that only examine a single final score, these process measures enable the detection of subtle preclinical signs of cognitive deficits and the classification of MCI subtypes, including single-domain or multiple-domain amnestic MCI (aMCI) and non-amnestic or dysexecutive MCI (naMCI) (33). Further, as suggested by Emrani et al. (34, 35), when brought to scale, digitally obtained, process-based measures could improve our ability to flag emergent neurodegenerative illness as early as possible.

Included in this new, emerging corpus of research are several reports where a digital version of the traditional paper and pencil Trail Making Tests have been used (14, 36–38). For example, Fellows et al. (39) recruited groups of healthy controls and a mixed group of neurologic/psychiatric patients. In addition to traditional time to completion, they tallied a number of process-based parameters, including pauses inside the target circles, pen lifts off the tablet, and time spent inside target circles. A series of regression analyses using other neuropsychological tests suggested that performance on the digital TMT-A was associated with tests measuring information processing speed. Similar regression analyses found total time to completion on the TMT-B was related to neuropsychological tests associated with inhibitory control, visual scanning, and visuospatial working memory. These researchers also obtained an in-clinic, performance-based assessment of medication management [i.e., the Medication Management Ability Assessment (MMAA)]; (13), and found that successful performance on the MMAA was related to digital TMT-B average pause duration, average time inside target circles, and average lift duration. Interestingly, similar analyses for TMT-A were not significant. The observation that digital TMT-B metrics are associated with an in-clinic assessment of medication management skills is both interesting and provocative. However, in clinical practice, IADL skills, such as medication management, are most often assessed with informant-rated questionnaires.

In the current research, a group of community-dwelling volunteers were assessed with an iPad version of the Trail Making Tests-Part B, and the Functional Activities Questionnaire (FAQ) (40), a commonly used informant-based IADL measure. Informant FAQ scores classified participants into three groups: unimpaired, subtle, and mild IADL disabilities. Between-group analyses were obtained to assess relationships between informant-based FAQ abilities and a panel of digital TMT-B process outcome measures. Moreover, factor analysis was undertaken to extract and better understand the neurocognitive constructs that underlie digital Trail Making Part-B test performance.

## Methods

### Participants

The sample consisted of 321 community-dwelling participants. These research participants were recruited for cognitive screening as part of an ongoing, multi-site, observational study of DCAs for AD clinical trial prescreening. The demographics for the entire sample were as follows: mean age = 70.21 ± 6.08; mean education = 15.85 ± 2.45; and mean MMSE = 28.14 ± 2.40; 56.00% female. Inclusion criteria consisted of completing the digital Trail Making Test-Part B (dTMT-B), being between 60 and 85, having a MMSE score of 20–30, and using English as the primary language. The exclusion criteria were extensive and based on underlying conditions, more information can be obtained by contacting the GAP consortium.^1^ All research participants provided written informed consent prior to participating in the study. Ethical approval was granted by the local IRB for each clinical site participating in the consortium.

### The digital Trail Making Test-Part B

**Figure 1:**
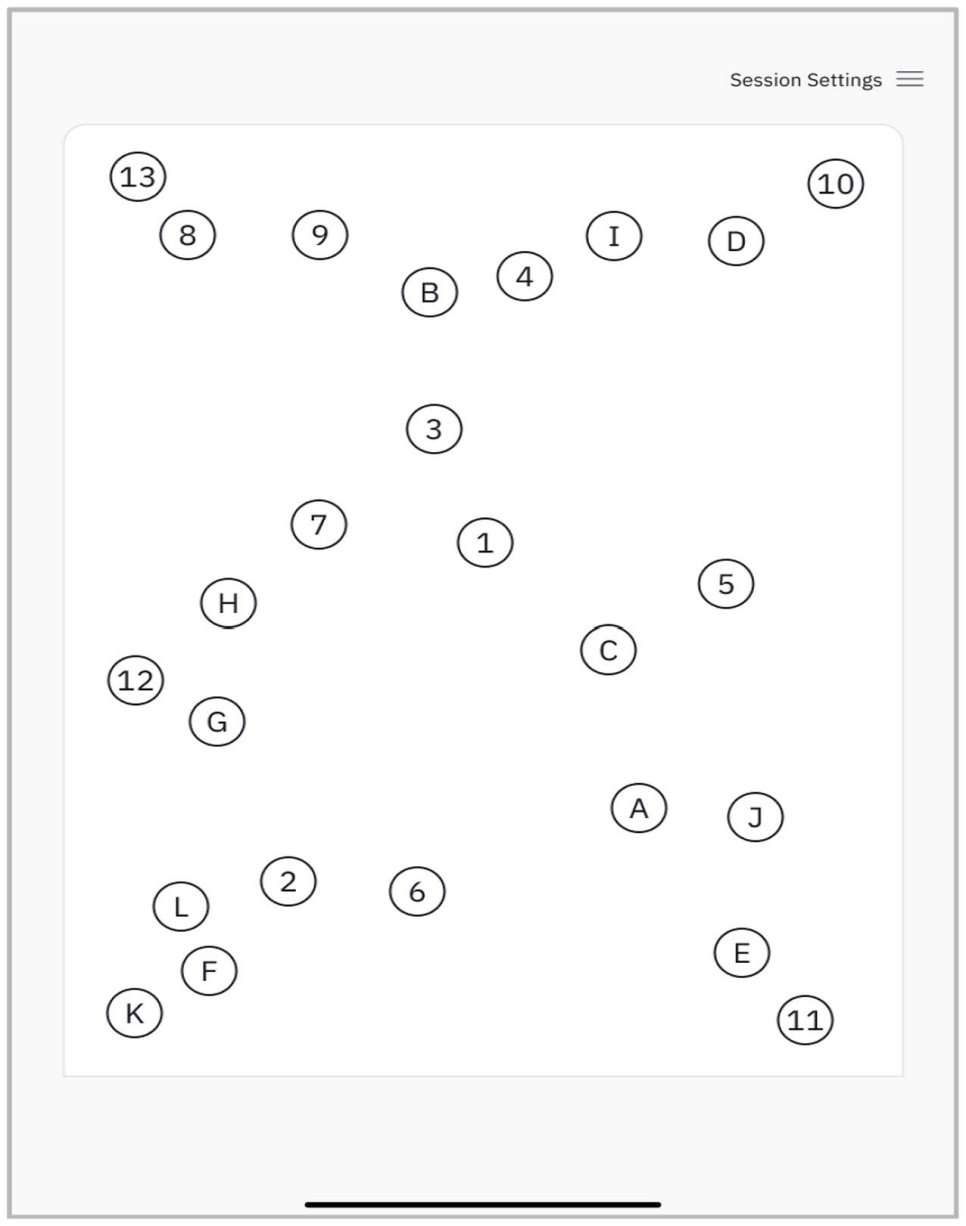
Example of the dTMT-B assessment screen detailing the target circles and their layout.

The dTMT-B is fully automated and administered using an iPad with a paired Apple Pencil. The participant is asked to connect circles alternating between numbers and letters in an ascending order (e.g., 1-A-2-B-3-C). The size of target circles and their layout mirror the TMT-B as described in Reitan and Wolfson (41) (Figure 1). The dTMT-B protocols were obtained by a trained psychometrist. If participants made an error by connecting a circle out of sequence, this line was erased from the user interface (UI), and the subject was queued to return to the previous circle and continue their stroke.

### dTMT-B outcome measures

A total of seven outcome measures were used in the current research. The iPad recorded the *total time to completion,* or the total amount of time necessary to complete the test in seconds. This outcome variable is analogous to the time to completion metric obtained using the traditional paper and pencil version of this test. In addition to total time to completion, a corpus of six comparatively new digitally produced process variables was used in the current research. Several of the outcome measures used in the current research have been described by other researchers (14, 39, 42).

*Total drawn strokes (total strokes):* This variable tallied the total number of drawn strokes necessary to complete the test, with a stroke defined as the time from when the stylus touched the screen to when it was raised from the screen.

*Duration inside target circles (circle pause duration):* This variable measured the time the stylus was in contact with the iPad while inside the target circles.

*Stylus lift duration (lift time):* This variable measured the time the pen was not in contact with or was lifted off the iPad.

*Line deviation (line deviation):* This variable provided a measure of the deviation from the optimally straight line drawn between the centers of successive circle targets (line deviation = total distance of a drawn path between two circles minus the shortest possible path between two circles, averaged for all paths made between target circles). A score of zero suggests no line deviation for a line drawn connecting successive target circles.

*Total distance (distance):* This variable measured the total length or distance of all drawing strokes used to complete the test.

*Mean drawing stroke velocity (stroke velocity):* This variable measured the average speed or velocity of individually drawn strokes connecting successive target circles (i.e., average velocity = path length between circles/path duration between circles, averaged for all paths made between circles).

### Functional activities questionnaire groups

The FAQ (range 0–30) is a pencil and paper, informant-rated questionnaire that assesses 10 IADL skills (e.g., routine financial operations, and shopping). All FAQ items are scored from 0 (normal ability) to 3 (dependent ability). Pfeffer et al. (40) suggested that a score cutoff of ≥9, i.e., dependence on three or more IADL activities, is indicative of IADL impairment. In the current research, participants rated by their study partner with a FAQ score of 0 were classified as unimpaired; participants with FAQ scores from 1 to 4 were classified with subtle IADL impairment; and participants rated with FAQ scores from 5 to 8 were classified with mild IADL impairment.

### Statistical analyses

Demographic and MMSE test performance was assessed with a series of one-way analyses of variance. Because of dTMT-B non-normality, non-parametric median tests were used to assess for FAQ between-group differences. The Bonferroni correction was applied to all between-group comparisons. A stepwise nominal regression analysis was obtained to assess how well the dTMT-B variables described above were able to classify participants into their respective groups, where the FAQ-unimpaired cohort was the reference group. Stepwise nominal regression was used because of the large number of predictor variables and the relatively modest sample size in some groups (43).

The corpus of dTMT-B variables described above was subjected to a factor analysis with direct oblimin rotation to extract underlying neurocognitive constructs. Exploratory direct oblimin, non-orthogonal rotation (SPSS) was used because of the presumed non-orthogonal interdependence between dTMT-B outcome measures (44). Finally, partial correlations between the resultant dTMT-B factors and each of the 10 FAQ items were obtained. These analyses were controlled for age, education, and sex. As described above, the Bonferroni correction was applied to these analyses.

## Results

### Demographic characteristics

Table 1 lists demographic and clinical information. No FAQ-group differences were found for age and education. The FAQ-unimpaired group obtained a marginally higher score on the MMSE than the FAQ-mild group (*F*[2, 318] = 4.15, *p* < 0.017; η^2^ = 0.025).

**Table 1:** Demographic and clinical information (means and standard deviations).

	FAQ-normal (*n* = 193)	FAQ-subtle (*n* = 103)	FAQ-mild (*n* = 25)	Significance
Age	69.80 (5.68)	70.75 (6.50)	71.20 (7.11)	ns
Education	15.69 (2.33)	16.05 (2.64)	16.24 (2.58)	ns
MMSE	28.39 (2.59)	27.94 (1.99)	27.04 (2.16)	FAQ-normal > FAQ-mild; *p* < 0.023
FAQ	00.00 (00.00)	2.06 (1.13)	6.44 (1.12)	
Gender (percent female)	56.00 percent			

ns, not significant; MMSE, Mini-Mental State Examination; FAQ, Functional Assessment Questionnaire.

### Between-group dTMT-B performance

Full statistics can be found in Table 2. Total time to completion was faster for FAQ-unimpaired participants compared to FAQ-subtle participants (*p* < 0.002); and there was a trend for slower total time to completion when FAQ-unimpaired participants were compared to FAQ-mild participants (*p* < 0.069). When compared to the FAQ-unimpaired group, pauses or time duration inside of the target circles was longer for FAQ-subtle participants (*p* < 0.002) and for FAQ-mild participants (*p* < 0.050). FAQ-unimpaired participants completed the dTMT-B assessment with fewer total number of stylus strokes than other groups (FAQ-unimpaired/FAQ-subtle, *p* < 0.005; FAQ-unimpaired/FAQ-mild, *p* < 0.016).

**Table 2:** Digital Trail Making Test—Part B (means and standard deviations).


	FAQ-normal (*n* = 193)	FAQ-subtle (*n* = 103)	FAQ mild (*n* = 25)	Significance
Total time to completion	110.15 (32.16)	123.87 (32.65)	124.81 (27.14)	U = 14.67; df = 2; *p* < 0.001
				Normal < subtle; *p* < 0.002
				Normal < mild; *p* < 0.069
Total strokes	7.08 (7.40)	8.65 (6.37)	9.48 (5.75)	U = 14.90; df = 2; *p* < 0.001
				Normal < subtle; *p* < 0.005
				Normal < mild; *p* < 0.016
Line deviation	0.059 (0.048)	0.066 (0.03)	0.076 (0.041)	U = 9.31; df = 2; *p* < 0.009
				Normal < subtle; *p* < 0.067
				Normal < mild; *p* < 0.044
Circle pause duration	115.47 (68.94)	146.80 (78.80)	131.35 (43.45)	U = 17.83; df = 2; *p* < 0.001
				Normal < subtle; *p* < 0.001
				Normal < mild; *p* < 0.050
Stroke drawing velocity	126.46 (30.40)	113.52 (28.88)	116.55 (24.97)	U = 14.37; df = 2; *p* < 0.001
				Normal > subtle; *p* < 0.001
				Normal = mild
Pen life duration	24.02 (19.09)	25.36 (16.40)	26.72 (13.49)	ns
Total drawing distance	10,095.31 (1,226.29)	10,387.02 (1,285.61)	10818.06 (1,811.52)	ns

ns, not significant; FAQ, Functional Assessment Questionnaire.

Stroke line deviation was greater for FAQ-mild participants compared to FAQ-unimpaired participants (*p* < 0.044); and there was a trend for greater line deviation when FAQ-subtle participants were compared to FAQ-unimpaired participants (*p* < 0.067). Individual pen stroke velocity, or the speed with which lines between successive circle targets were drawn, was faster for FAQ-unimpaired participants compared to FAQ-subtle participants (*p* < 0.001). Finally, the effect of the FAQ group on the total distance of all pen strokes was significant (p < 0.002), suggesting greater drawing distance produced by FAQ-subtle and mild participants compared to FAQ-unimpaired participants; however, after the Bonferroni correction was applied, these comparisons were no longer statistically significant. No between-group differences were observed for pen lift time.

### Nominal regression

The corpus of seven dTMT-B measures described above was analyzed with a stepwise nominal regression (forward entry), where the FAQ-unimpaired cohort was the reference group. Only pause duration or time spent inside the target circles entered the model (*X^2^ =* 20.92, *p* < 0.001), and was able to classify participants into their respective groups (FAQ-unimpaired/FAQ-subtle, Wald = 17.44, *p* < 0.001; FAQ-unimpaired/FAQ-mild, Wald = 5.91, *p* < 0.015).

### Factor analysis

Two separate two-factor solutions were obtained. In the first analysis, only the six new dTMT-B process variables were included. This analysis was undertaken to extract neurocognitive constructs *exclusively* related to the comparatively new, digitally defined process metrics. This analysis yielded a two-factor solution (77.84% variance explained). Factor 1 (48.96% variance explained) appears to be related to impaired, rudimentary motor/neurocognitive operations, i.e., a greater number of pen strokes, a greater line deviation, longer pen lift time, and a greater total drawing distance. On the other hand, factor 2 (28.88% variance explained) appears to describe relatively intact, more efficient motor/neurocognitive operations, i.e., faster velocity or speed of drawing between successive target circles, and shorter pause duration or time spent inside the target circles (Table 3).

**Table 3:** Digital trails: factor analysis (excluding total time to completion).

	Factor 1	Factor 2
Total strokes	0.965	0.007
Line deviation	0.957	0.226
Circle duration	0.144	−0.938
Pen lift duration	0.698	−0.081
Total distance	0.640	−0.155
Mean stroke velocity	0.093	0.972
Eigen value/percent variance	2.93	1.73
	48.96%	28.88%

The second factor analysis included the six new dTMT-B process variables, along with the traditional total time to completion metric. As displayed in Table 4, the composition of the six process variables was not significantly changed. However, the traditional total time to completion variable is cross-loaded between the two factors. Thus, on factor 1 (rudimentary motor/neurocognitive operations), there was a *positive* factor loading to suggest that the comparative impairment illustrated by the dTMT-B process variables was associated with a *slower* total time to completion. However, on factor 2 (efficient motor/neurocognitive operations), there was a *negative* factor loading for total time to completion to suggest *faster* total time to completion.

**Table 4:** Digital trails: factor analysis (including total time to completion).

	Factor 1	Factor 2
Line deviation	0.970	0.248
Circle duration	0.068	−0.945
Pen lift duration	0.695	−0.165
Total distance	0.626	−0.131
Mean stroke velocity	0.174	0.977
Total time to completion	0.419	−0.779
Total strokes	0.962	0.012
Eigen value/percent variance	3.69	1.89
	52.84%	27.12%

### Partial correlations: FAQ items and factor analysis variables

Using the factor analysis comprised of only the six new dTMT-B process metrics, new variables were created, estimating factor score coefficients with a mean of 0 and variance equal to the squared multiple correlation between the estimated factor scores and the true factor values (SPSS v29). As described above, these factors appear to be related to impaired relatively rudimentary motor/neurocognitive abilities (factor 1) and efficient motor/neurocognitive operations (factor 2). Correlation analyses were obtained between these factor analytic-derived variables and the 10 FAQ items.

A negative correlation was obtained, suggesting that *greater* impairment in rudimentary motor/neurocognitive operations (factor 1) was associated with *greater* dependence on shopping for clothes, household necessities, or groceries (r = −0.121, *p* < 0.029). This is juxtaposed with positive correlations between more efficient motor/neurocognitive operations (factor 2) and relatively intact independence for shopping for clothes, household necessities, or groceries (r = 0.119, *p* < 0.030), keeping track of current events (r = 0.200; *p* < 0.001), and paying attention, understanding, and discussing TV, books, and magazines (r = 0.148, *p* < 0.007). Only correlations between factor 2 and keeping track of current events and paying attention, understanding, and discussing TV, books, and magazines persisted after the Bonferroni correction was applied. Full statistics can be found in Table 5.

**Table 5:** dTMT-B and FAQ items: partial correlation.

	dTMT-B Factor 1	dTMT-B Factor 2
Paying bills	−0.095	0.087
Assembling records	−0.031	0.002
Shopping independently	**−0.121; p < 0.029**	**0.119; p < 0.030**
Games of skill and hobbies	−0.034	0.105
Making coffee	−0.063	0.101
Preparing a balance meal	−0.080	0.103
Keeping abreast of current events	+0.016	**0.200; p < 0.001**
Understanding TV and books	−0.007	**0.148; p < 0.007**
Memory for appointments, family occasions, medication	+0.016	−0.072
Independent travel	−0.064	0.071

FAQ, Functional Assessment Questionnaire. Bold values are stylistically meaningful.

## Discussion

The current research examined how possible dysexecutive behavior using a digital version of the Trail Making Test-Part B might be associated with comparatively subtle-to-mild informant-based, IADL activities using the Functional Assessment Questionnaire (FAQ). Published research using the FAQ suggests that a score ≥ 9 is a reasonable cut score to identify individuals with IADL problems. Nonetheless, prior research suggests that even subtle IADL decline can be accompanied by neuropsychological difficulty (45). For this reason, the FAQ was used to characterize groups with unimpaired, subtle, and mild informant-based IADL difficulty.

In addition to time to completion, the traditional metric used to define impairment with the paper and pencil version of the Trail Making Test-Part B, a panel of six, comparatively new novel process-based measures (14, 39) was examined in relation to FAQ groups. A wide number of between-group differences were revealed. As compared to the FAQ-unimpaired group, other FAQ groups were slower to complete the entire test, were slower at drawing lines that connected successive test stimuli, spent more time paused inside target circles before proceeding to the next test stimuli, required more pen strokes to complete the test, and exhibited difficulty drawing straight lines connecting successive test stimuli.

The stepwise nominal regression analysis was conducted to assess how well the traditional time to completion, or the new digital TMT-B metrics could classify patients into their respective groups. Interestingly, only pause duration or time spent inside the target circles entered the model. These results are compelling. Nonetheless, as seen in Supplementary Table 1, all of these metrics are highly correlated with each other. Thus, the results of this analysis need to be interpreted with caution. Additional research with similar analyses needs to be undertaken to assess how well these new digital metrics operate for patient classification.

A factor analysis of these six new dTMT-B process-based variables suggests the presence of two underlying neurocognitive constructs. The production of dTMT-B protocols with greater numbers of pen strokes, difficulty drawing straight lines between successive test stimuli, greater time spent with the pen lifted from the iPad, and greater total drawing distance when the length of all pen strokes was tallied could suggest impairment involving a combination of comparatively rudimentary motor/neurocognitive operations. By contrast, other novel process-based dTMT-B measures, i.e., greater speed or velocity drawing pen strokes between successive test circles and less pause time when the pen is inside target circles, suggest comparatively intact motor/neurocognitive operations.

Some validity for these observations is supported when these factor analytic-derived metrics were correlated with FAQ test items. As described above, what appears to be compromised dTMT-B motor/neurocognitive operations were associated with a statistical trend suggesting greater informant-based impairment on selected FAQ items such as the ability to shop independently, an activity that is heavily reliant on mobility. However, more robust correlations were obtained between dTMT-B motor/neurocognitive operations and selected intact informant-based FAQ items requiring critical higher mental activities such as keeping up with current events and paying attention to complex media sources. Positive findings on the dTMT-B, as described above, could reveal a nascent illness along with possible treatment. Thus, clinically, the data described above suggest that the dTMT-B is an excellent test that could be used in both primary and specialty care to screen for mild or even subclinical IADL disabilities.

A closer examination of the factor analytic solutions described above suggests that an even wider variety of motor/neurocognitive operations appear to underlie performance on the dTMT-B. For example, motor problems or subtle disabilities in controlling the pen when drawing could be responsible for dTMT-B protocols with extraneous pen strokes, poorly drawn lines connecting successive target circles, and greater total drawing distance. Subcortical vascular disease could underlie these problems. Indeed, Davoudi et al. (46) described similar graphomotor impairment in their analysis of digital clock drawings produced by dementia patients with evidence of MRI subcortical white matter alterations.

Greater time spent with the pen lifted from the iPad could be associated with additional problems, including deficits revolving around visual scanning for successive target items and/or struggling to maintain the assigned mental set for this test, i.e., *to draw a line alternating between numbers and letters.* The prior studies of Du et al. (14) and Fellows et al. (39) tend to support this supposition. In research examining participants from the Framingham Heart Study, De Anda-Duran et al. (47) found that paper and pencil Trail Making Part B pen lifts was associated with subtle but statistically significant MRI gray and white matter alterations involving frontal, parietal, and temporal lobe brain regions—regions known to be involved in working memory functions [see (48)]. Moreover, prior research using the DCTclock™ has revealed the presence of intra-component or decision-making latencies (49) [see (26) for a review]. This behavior refers to measurable pauses or latencies between, say, drawing the clock face and the next stroke. Dion et al. (30) and Libon et al. (49) found that slower clock drawing intra-component latencies were associated with worse or reduced neuropsychological test performance.

The presumptive *‘visual scanning’* problems associated with the dTMT-B could be due to several problems. For example, Shi et al. (50) recently summarized a growing body of research suggesting an association between cerebral and retinal vasculopathy and cognitive decline in AD and MCI. These authors also noted an association between retinal vascular platelet-derived growth factor receptor-β (PDGFRβ) expression and greater pericyte loss along with retinal vascular amyloidosis and cerebral amyloid angiopathy in MCI and AD patients. Nishioka et al. (51) used diffusion tensor imaging (DTI) technology to examine visual pathways in patients with AD, MCI, and healthy controls and found increasing total diffusivity and radial diffusivity along with reductions in fractional anisotropy in optic nerves in AD and MCI patients. The changes seen in visual pathways mirrored changes in the splenium of the corpus callosum and were thought to be due to white matter alterations. All of these problems could result in slower total time to completion and impairment of other dTMT-B variables, as described above.

On the other hand, greater velocity or speed in drawing lines between successive target circles and less pause time when the pen is inside the target circles suggest participants were able to exercise inhibitory control (39) and marshal the necessary neurocognitive resources to maintain the assigned mental set associated with this test. Thus, as seen in Table 4, the total time to completion is faster. All of these data provide empirical support for what we know, i.e., that the variety or multitude of neurocognitive operations associated with successful Trail Making Part-B performance is very sensitive to the presence of brain illness. Moreover, IADL activities, as queried in the FAQ, are also associated with diverse and multiple underlying cognitive abilities. Therefore, it is perhaps not surprising that even minimal IADL alterations as defined in the current research are associated with a range of de-railed behavior when assessed with the dTMT-B.

The current research is not without limitations. First, no information regarding handedness was obtained in the current research. The degree of sinistrality could have affected the results reported above. Second, errors that may have been made on the dTMT-B, data from the dTMT-B practice portion of the test, and data from the companion digital Trail Making-Part A test condition were not available for analysis. As reported by other researchers (14, 39), these data need to be presented in order to obtain a fuller appreciation of the relations between FAQ-defined IADL activities and neuropsychological problems using the dTMT-B. Third, we acknowledge that the decision to create subtle and mildly impaired FAQ groups, as described above, is somewhat arbitrary and may have affected the results as reported. Moreover, future research regarding IADL abilities and the dTMT-B should be explored using a range of methods. Similarly, data from additional clinical groups, such as patients with movement disorders, would be useful.

Finally, an issue not explored in the current research is how the time-based and graphomotor metrics used in the current research change as a function of total time to completion. In previous research with patients diagnosed with Alzheimer’s disease/vascular spectrum dementia (52) and memory clinic patients characterized with various MCI subtypes (53), patients with vascular dementia and dysexecutive MCI, respectively, tend to display disproportionately greater dysexecutive impairment on the latter test epochs. These types of process-based measures, in conjunction with other process-based measures used by other research groups (14, 39) and in the current research, could increase the sensitivity of the dTMT-B to flag individuals with emergent neurodegenerative illness. Given the recent availability of disease-modifying medication to treat MCI and mild Alzheimer’s disease, there is increased urgency to develop effective and sensitive neuropsychological tests to screen for these disorders.

Despite these limitations, the current research has several strengths. For example, the data described in the current research replicates past dTMT-B research findings. Thus, in the current research and in the prior report of Fellows et al. (39), meaningful dTMT-B information regarding drawing pauses, time inside target circles, pen lifts, and time between target circles is described. The current research expands upon prior dTMT-B research in that several additional dTMT-B variables, including stroke velocity, total strokes, and total distance, are now added to the portfolio of dTMT-B outcome variables. Moreover, the results from both factor analyses suggest that dTMT-B behavior might be used to define important neurocognitive constructs related to neurodegenerative illness.

In sum, the current research provides an excellent example of what can be learned when traditional paper and pencil neuropsychological tests are coupled with new digital assessment technology.

## Data availability statement

The datasets presented in this article are not readily available because data is not available at the current time. Requests to access the datasets should be directed to the GAP Consortium (https://globalalzplatform.org/).

## Ethics statement

The studies involving humans were approved by the GAP Consortium (https://globalalzplatform.org/). The studies were conducted in accordance with the local legislation and institutional requirements. The participants provided their written informed consent to participate in this study.

## Author contributions

DL: Conceptualization, Formal analysis, Investigation, Methodology, Validation, Writing – original draft, Writing – review & editing. RS: Conceptualization, Formal analysis, Methodology, Validation, Writing – original draft, Writing – review & editing. ST: Conceptualization, Formal analysis, Methodology, Validation, Writing – review & editing. AJ: Conceptualization, Writing – review & editing. DS: Writing – review & editing. CP: Conceptualization, Methodology, Writing – review & editing. ML: Conceptualization, Methodology, Writing – review & editing. AP-L: Writing – review & editing.

## Footnotes

^1^ https://globalalzplatform.org/
