# Supplementary material for: Dysexecutive difficulty and subtle everyday functional disabilities: the digital Trail Making Test
Source: Front Neurol. 2024 Apr 3;15:1354647. doi: 10.3389/fneur.2024.1354647 (PMC11021769; doi:10.3389/fneur.2024.1354647)
Supplement: Supplementary file 1 [file Data_Sheet_1.docx]

***Supplementary Material***

Dysexecutive Difficulty and Subtle Everyday Functional Disabilities:

The Digital Trail Making Test

**Correspondence:** Corresponding Author: libon@rowan.edu

# Trial Inclusion/Exclusion Criteria

**Inclusion Criteria:**

1. Participants and/or a legally authorized representative (LAR) must provide signed and dated informed consent and authorization to use personal health information in accordance with local and national guidance and regulations;
2. Male or female 50 to 90 years of age (inclusive) at the time of consent;
3. Participants must have a Mini-Mental State Exam (MMSE) score of 20 to 28 inclusive;
4. Progressive cognitive complaints must be reported by participant or caregiver;
5. Participants must be willing to comply with all procedures as outlined in the informed consent, including blood sampling, genetic testing, and storage of biospecimens for future research;
6. Fluency in the language of the tests used at the site;
7. Participants must be interested in participating in clinical research.

**Exclusion Criteria:**

1. Participants who, in the opinion of the Investigator, have serious or unstable medical conditions that would prohibit their completion of all prescreening procedures and data collection;
2. Participants who are currently enrolled in another clinical study.
3. Participants who have serious or unstable medical conditions that would likely preclude their participation in an interventional research trial;
4. Participants who have reported or have a known negative amyloid PET scan in the past 24 months;
5. Participants with history of stroke within 6 months of prescreening;
6. Participants with an uncontrolled seizure disorder, unexplained blackouts, OR history of a seizure within 6 months (subjects with a history of pediatric febrile seizure, benign Rolandic epilepsy may participate);
7. Participants with a history or evidence of a malignancy within the 2 years prior to prescreening. Subjects with indolent malignancies (e.g., basal cell carcinoma or squamous cell carcinoma) or malignancies considered to be cured and not actively treated with anti-cancer therapy or radiotherapy are permitted to enroll;
8. Participants with known or suspected alcohol or drug abuse or dependence within 2 years of prescreening;
9. Participants with a reported suicidal attempt within 2 years of prescreening), or any unstable psychiatric symptoms (e.g., uncontrolled depression);
10. Participants who have participated in a clinical trial of any potential disease modifying AD treatment and received active drug within 6 months prior to prescreening;
11. Participants who have any neurological disorder affecting the central nervous system, other than AD, that may be contributing to cognitive impairment (e.g., Parkinson's disease, other dementias, multiple concussions or seizures) as deemed significant by the Investigator;
12. Participants with known history of hepatitis C virus, hepatitis B virus, human immunodeficiency virus (HIV) or other immunodeficiencies;
13. Participants that have previously been consented to this protocol;
14. Participants with a hypersensitivity to mAb treatments, protein derived from a mAb, or immunoglobulin therapy;
15. Participants with allergies to diphenhydramine, epinephrine, and methylprednisolone;
16. Participants who are direct employees or family members of direct employees of the participating investigators' sites;
17. Participants who are direct employees of the Sponsor;
18. Participants who, in the opinion of the Investigator, are unable to complete cognitive testing due to inadequate visual or auditory acuity.
