# Supplementary material for: Dysexecutive difficulty and subtle everyday functional disabilities: the digital Trail Making Test
Source: Front Neurol. 2024 Apr 3;15:1354647. doi: 10.3389/fneur.2024.1354647 (PMC11021769; doi:10.3389/fneur.2024.1354647)
Supplement: Supplementary file 2 [file Table_1.docx]

| **Supplements Table 1: Correlation between dTMT-B Variables** | | | | | | | |
| --- | --- | --- | --- | --- | --- | --- | --- |
|  | Duration | Total  Strokes | Hit Duration | Lift Duration | Line Deviation | Total Distance | Mean Velocity |
| Duration | 1 |  |  |  |  |  |  |
| Total  Strokes | 0.554;  p< 0.001 | 1 |  |  |  |  |  |
| Hit  Duration | -0.856;  p< 0.001 | -0.303;  p< 0.001 | 1 |  |  |  |  |
| Lift  Duration | 0.704;  p< 0.001 | 0.625;  p< 0.001 | -0.235;  p< 0.001 | 1 |  |  |  |
| Line  Deviation | 0.331;  p< 0.001 | 0.994;  p< 0.001 | -0.096;  ns | 0.491;  p< 0.001 | 1 |  |  |
| Total  Distance | 0.456;  p< 0.001 | 0.520;  p< 0.001 | -0.413;  p< 0.001 | 0.290;  p< 0.001 | 0.473;  p< 0.001 | 1 |  |
| Mean  Velocity | 0.717;  p< 0.001 | 0.127;  p< 0.012 | -0.859;  p< 0.001 | 0.167;  p< 0.001 | -0.075;  ns | 0.061;  ns | 1 |
